# New Appliances and Things Medical

**Published:** 1899-10-28

**Authors:** 


### NEW APPLIANCES AND THINGS MEDICAL.

**[We shall be glad to receive, at our Office, 28 & 29, Southampton Street, Strand, London, W.C., from the manufacturers, specimens of all new preparations and appliances which may be brought out from time to time.]**

### NEW TONGUE DEPRESSOR.

(Maw, Son, and Thompson, 7 12, Aldersgate Street, London, E.C.)

This new tongue depressor is made of plated metal, with a well-shaped tongue-piece roughened on the lower surface to ensure a good grip of the tongue. The handle is specially shaped with a double curvature inclining chiefly towards the left, so that by holding with the left hand plenty of room is left on the right side, where there is nothing to obstruct the view or interfere with manipulation with instruments, &c. For cauterising and other minor operations on the palate, tonsils, and pharynx it will be found exceedingly useful.

### LAGER BEER.

(Allsopp's Brewery, Burton-on-Trent.)

Allsopp's brewery is now producing a lager beer of very superior quality. We have received samples of two varieties, the light and the dark, corresponding respectively to what are known by the terms Pilsener and Münchener. From a medical standpoint much interest attaches to this vast enterprise in the production of a lager beer in this country which is equal to the best beer of its sort brewed on the Continent, and at the same time can be confidently relied upon as being absolutely pure in a chemical and hygienic sense. In the first place the manufacturers guarantee the sole employment of hops and malt, and in the second place the most scrupulous care is taken to prevent accidental contamination with foreign germs or undesirable ferments. The system on which the beer is brewed is known as the Pfaudler vacuum process. By this method the primary and secondary fermentative processes are effected at a single operation, the time is enormously reduced, and the vessels used being glass-lined can always be kept in a condition of scientific cleanliness. To sum up in a few words, the beer brewed by Messrs. Allsopp on the Pfaudler system is a lager beer at least equal to what can be obtained on the Continent. It is a cheap beer because it can be brewed in a comparatively short space of time, and lastly, it is a pure beer, the result of fermentative processes which are under absolute control. No accidental ferments can possibly find their way into the brew to spoil the flavour or interfere with the chemical purity of the products of alcoholic fermentation. For the latter reason Allsopp's lager beer can be safely recommended by the medical profession, where a pure alcoholic beverage, the result of malt fermentation, combined with hop flavouring, are considered to be of advantage.

### DYLISSIA DENTIFRICE AND TOILET ARTICLES.

(Durant and Co., 19, Mount Pleasant, London, E.C.)

Dylissia dentifrice appears a carefully manufactured and harmless preparation for the teeth. It has an excellent stimulating action on the gums, and is particularly agreeable to use. It cleanses the teeth rapidly and well, and is in no way injurious to the enamel. The toilet powder of the same "Dylissia" brand is made in three tints and perfumed with otto of roses. It is an exceedingly fine and soft powder, non-irritating to the skin. Other powders by the same firm are worthy of attention, namely, Dylissia Fuller's earth, which is a particularly fine sample of this invaluable nursery requisite. Various creams for nursery and toilet use also belong to the same series, and, as far as we have been able to examine them, they appear carefully prepared, of good quality, and free from injurious ingredients; in fact, reliable and safe articles for general use.

### COURVOISIER'S COGNAC.

(Bottled by Marshall and Elvy, 45, New Oxford Street, London, W.C.)

This high-class cognac received the gold medal at the Paris Exposition in 18[illegible]9, and the Algiers Exposition gold medal in 1889. We have received two samples of this brandy, eight years and twenty years old respectively. The latter is a remarkably fine liqueur brandy, possessed of the finest aromatic ethers. Although, perhaps, too much a brandy "de luxe" for ordinary medicinal use, in exceptional cases, where expense is of no consideration, it will be found most acceptable. The first-named brandy, guaranteed eight years old, is a beautifully soft and mellow spirit of delicious taste. Invalids and others will find it admirably suited for cases in which the stimulating qualities of a real cognac brandy are a desideratum.

